# A phase I trial of riluzole and sorafenib in patients with advanced solid tumors: CTEP #8850

**DOI:** 10.18632/oncotarget.28403

**Published:** 2023-04-10

**Authors:** Kristen R. Spencer, Daniella E. Portal, Joseph Aisner, Mark N. Stein, Jyoti Malhotra, Weichung Shih, Nancy Chan, Ann W. Silk, Shridar Ganesan, Susan Goodin, Murugesan Gounder, Hongxia Lin, Jiadong Li, Robert Cerchio, Christina Marinaro, Suzie Chen, Janice M. Mehnert

**Affiliations:** ^1^Rutgers Cancer Institute of New Jersey, Rutgers, The State University of New Jersey, New Brunswick, NJ 08903, USA; ^2^Department of Medicine, Division of Medical Oncology, Rutgers Robert Wood Johnson Medical School, Rutgers University, Piscataway, NJ 08854, USA; ^3^Department of Biostatistics, School of Public Health, Rutgers, The State University of New Jersey, Piscataway, NJ 08854, USA; ^4^Dana-Farber Cancer Institute, Boston, MA 02215, USA; ^5^Susan Lehman Cullman Laboratory for Cancer Research, Ernest Mario School of Pharmacy, Rutgers, The State University of New Jersey, Piscataway, NJ 08854, USA; ^6^Department of Pharmacology, Rutgers Robert Wood Johnson Medical School, Rutgers, The State University of New Jersey, Piscataway, NJ 08854, USA; ^7^Department of Medicine, New York University Grossman School of Medicine, Perlmutter Cancer Center of NYU Langone Health, NY 10016, USA

**Keywords:** GRM1, riluzole, sorafenib, phase I, clinical trial

## Abstract

Background: Overexpression of metabotropic glutamate receptor 1 (GRM1) has been implicated in the pathogenesis of multiple cancers. Riluzole, an inhibitor of glutamate release, showed synergistic antitumor activity in combination with the multi-kinase inhibitor sorafenib in preclinical models. This phase I trial identified the toxicity profile, dose-limiting toxicities, maximum tolerated dose (MTD), and pharmacokinetic and pharmacodynamic properties of riluzole combined with sorafenib in patients with advanced cancers.

Patients and Methods: Patients with refractory solid tumors were enrolled utilizing a 3+3 dose-escalation design. Riluzole was given at 100 mg PO BID in combination with sorafenib, beginning at 200 mg PO daily and escalating in 200 mg increments per level in 28-day cycles. Restaging evaluations were performed every 2 cycles.

Results: 35 patients were enrolled over 4 dose levels. The MTD was declared at dose level 3 (riluzole: 100 mg PO BID; sorafenib: 400 mg AM/200 mg PM). Pharmacokinetic analyses did not reveal definitive evidence of drug-drug interactions. Consistent decreases in phospho-forms of ERK and AKT in tumor tissue analyses with accompanying decrease in GRM1 expression and increase in pro-apoptotic BIM suggest target engagement by the combination. Best responses included a partial response in 1 (2.9%) patient with pancreatic acinar cell carcinoma with a KANK4-RAF1 fusion, and stable disease in 11 (36%) patients.

Conclusion: Combination therapy with riluzole and sorafenib was safe and tolerable in patients with advanced solid tumors. The partial response in a patient with a RAF1 fusion suggests that further exploration in a genomically selected cohort may be warranted.

## INTRODUCTION

Signaling through glutamate metabotropic receptors (GRMs) has been implicated in the pathogenesis of central nervous system tumors [1–4], and breast, prostate, and melanoma tumors [5–7]. High expression of GRMs is seen in many tumor cell lines and subsets of human tumors [8, 9]. GRM1 activation induces downstream MAPK and PI3K-AKT-mTOR signaling [10], pathways known to be aberrantly activated in multiple cancers, particularly melanoma [11, 12]. It has also been demonstrated that GRM1 promotes a metabolic phenotype that supports increased glutamate production and autocrine glutamatergic signaling leading to downstream mitogenic signaling; this phenotype may be pharmacologically targeted to reduce glutamate bioavailability and interfere with tumor growth [13].

It has previously been demonstrated that ectopic expression of GRM1 in melanocytes leads to transformation of melanocytes *in vitro* and tumorigenesis *in vivo,* and that GRM1 is strongly expressed in a majority of human melanoma cell lines and biopsies, but not in normal melanocytes [7]. Riluzole, an inhibitor of glutamate release that is FDA approved for the treatment of Amyotropic Lateral Sclerosis (ALS), reduced tumor cell proliferation *in vitro* and decreased tumor growth *in vivo* [7, 14, 15]. Based on these observations, a 12-patient, pilot phase 0 trial of riluzole treatment was conducted in patients with stage III and IV melanoma prior to surgical resection [16]. One-third of the patients treated with the highest dose of riluzole used in clinical practice (100 mg PO BID) for 14 days showed evidence of suppression of pERK and pAKT signaling in paired tumor biopsies and an associated decrease in metabolic activity on post-treatment PET-CT scans. However, a subsequent phase II study of riluzole in patients with stage III unresectable or stage IV melanoma showed no responses in the first 13 patients and accrual was terminated [17].

Given that activation of multiple simultaneous pathways plays a key role in the growth of human melanomas [15, 18, 19], riluzole was screened in combination with second agents, including other kinase inhibitors with known activity in melanoma, using MTT assays and 3D cultures [20, 21]. Given the pathogenesis of melanoma includes several complex signaling mechanism including but not limited to MAPK and PI3K/AKT, both PLX4720/PLX4032 and sorafenib were investigated as potential agents. PLX4720/PLX4032 is a mutant B-RAF inhibitor, also known as vemirafenib, and sorafenib is a multi-kinase that inhibits both C-RAF and B-RAF and also affects signaling through downstream VEGF pathways. A combination of riluzole with sorafenib resulted in synergistic inhibition of growth and survival of BRAF wildtype and BRAF mutant GRM1 positive cell lines, both *in vitro* and in mouse xenograft models [21]. The antitumor efficacy of this combination was superior compared to PLX4720/PLX4032 [20, 21]. Based on these results, we conducted a phase I trial of riluzole and sorafenib in patients with advanced solid tumors to characterize the safety profile and determine the maximum tolerated dose (MTD) of the combination, with a planned expansion cohort in patients to evaluate clinical response and pharmacodynamic markers of GRM1, MAPK and PI3K pathway signaling.

## RESULTS

### Patient characteristics

Thirty-five patients with advanced solid tumors were enrolled from February 2011 to April 2017. Demographic and clinical characteristics of enrolled patients are listed in Table 1. The most common primary tumor sites were melanoma (*N* = 10) and colorectal cancer (*N* = 7). Fifty-four percent of patients (*N* = 19) had ≥3 prior lines of treatment, with the most common being cytotoxic systemic chemotherapy (*N* = 22; 63%), immunotherapy (*N* = 9; 26%) and targeted therapy (*N* = 2; 6%). None of the 35 patients had prior therapy with drugs targeting MAPK signaling, as no patients had mutations considered actionable by available therapies at the time of study enrollment.

**Table 1:** Patient demographics

Demographics	Patients (*n* = 35)
**Gender, *n* (%)**	
Male	16 (46)
Female	19 (54)
**Race, *n* (%)**	
White	27 (77)
Black or African American	7 (20)
≥1 Race	1 (3)
**Ethnicity, *n* (%)**	
Hispanic	4 (11)
Non-Hispanic	31 (89)
**Age, years**	
Median (range)	67 (29–91)
**ECOG Performance Status**^1^	
0	13 (37)
1	16 (46)
2	6 (17)
**Primary Tumor Site, *n* (%)**	
Cartilage	1 (3)
Cervical	1 (3)
Colorectal	7 (20)
Endometrial	1 (3)
Esophageal	1 (3)
GIST^2^	1 (3)
Melanoma	10 (29)
Mesothelioma	1 (3)
NSCLC^3^	3 (9)
Ovarian	3 (9)
Pancreatic	1 (3)
Peripheral Nerve Sheath Tumor	1 (3)
Sarcoma	2 (6)
Urothelial	2 (6)
**Stage, *n* (%)**	
IIIB	1 (3)
IIIC	6 (17)
IV	27 (77)
Unstaged, Refractory (Mesothelioma)	1 (3)
**Prior Therapy, *n***	
Prior Systemic Therapy	
1	6 (17)
2	8 (23)
≥3	19 (54)
Prior Surgery	22 (63)
Prior Radiation Therapy	17 (49)

Abbreviations: ^1^ECOG: Eastern Cooperative Oncology Group; ^2^GIST: Gastrointestinal Stromal Tumor; ^3^NSCLC: Non-Small Cell Lung Cancer.

### Dose escalation and determination of MTD

A summary of the four DLTs can be seen in Table 2. The first DLT was grade 3 maculopapular rash at dose level (DL) 2. None of the subsequent 3 patients enrolled on this DL experienced a DLT. Two of three patients at DL 4 experienced DLTs (grade 3 palmar plantar erythrodysesthesia and grade 3 maculopapular rash). Per protocol, an additional 3 patients were enrolled at the previous dose level (DL 3). One of these patients experienced a DLT of grade 3 hypophosphatemia, therefore the MTD was declared to be DL 3 (riluzole 100 mg PO BID and sorafenib 400 mg PO q AM/200 mg PO q PM). An expansion cohort of patients with tumors amenable to biopsy was subsequently opened at DL 3.

**Table 2:** Dose levels and dose limiting toxicities (DLTs)

Dose level	Dosing	# of patients	# of patients with DLT	DLT event (Grade)
1	Riluzole: 100 mg BID	4	0	N/A
	Sorafenib: 200 mg qd			
2	Riluzole: 100 mg BID	6	1	Maculo-papular rash (Grade 3)
	Sorafenib: 200 mg BID			
3	Riluzole: 100 mg BID	9	1	Hypophosphatemia (Grade 3)
	Sorafenib: 400 mg q AM, 200 mg q PM			
4	Riluzole: 100 mg BID	3	2	Palmar-plantar erythrodysesthesia syndrome (Grade 3) Maculo-papular rash (Grade 3)
	Sorafenib: 400 mg q AM, 400 mg q PM			
Expansion	Riluzole: 100 mg BID	13	0	N/A
	Sorafenib: 400 mg q AM, 200 mg q PM			

### Toxicity

All 35 patients received ≥1 dose of treatment and were evaluable for toxicities. The median number of cycles on treatment was 2 (range: 1–10). Adverse events felt probably, possibly or definitely related to sorafenib, riluzole, or the combination are displayed in Supplementary Table 1. Fatigue (*N* = 3), nausea (*N* = 9), and diarrhea (*N* = 6) were the most common adverse events related to the combination. Grade ≥3 related toxicities attributed to sorafenib, riluzole, or the combination are displayed in Table 3. Grade ≥3 related toxicities attributed to both drugs included fatigue (*N* = 1), anorexia (*N* = 1), maculopapular rash (*N* = 1), neutropenia (*N* = 1), and lymphopenia (*N* = 1).

**Table 3:** Grade ≥3 drug related adverse events

AE	Attribution		
	Both S + R	S (only)	R (only)
Fatigue	1	4	1
Anorexia	1	0	0
Maculopapular Rash	1	4	0
Lymphocyte Count Decrease	1	6	0
Neutrophil count decrease	1	0	0
White Blood Cell Decreased	0	2	0
Alkaline Phosphatase Increase	0	2	0
Arthralgia	0	1	0
Hypertension	0	1	0
Hypokalemia	0	1	0
Hyponatremia	0	2	0
Hypophosphatemia	0	4	0
Lipase Increase	0	2	0
Palmar-plantar erythrodysesthesia syndrome	0	1	0
Anemia	0	1	0
Cardiac troponin I increased	0	1	0
Congestive Heart Failure	0	1	0
Myocardial infarction	0	1	0
Pruritus	0	1	0
Wound complication	0	1	0
Abdominal Pain	0	0	1

Toxicities attributed to sorafenib by the treating investigator were observed in 80% (*N* = 28/35) of patients and included fatigue in 13 patients (4 of which were grade ≥3), maculopapular rash in 11 patients (4 of which were grade ≥3), palmar-plantar erythrodysesthesia (1 of which was grade ≥3), hypophosphatemia in 11 patients (4 of which were grade ≥3), and lymphopenia in 10 patients (6 of which were grade ≥3). Far fewer patients experienced toxicities attributed to riluzole alone (*N* = 17/35) and these included: dry skin (*N* = 3), ataxia (*N* = 2), insomnia (*N* = 2), aspartate aminotransferase increase (*N* = 2), weight loss (*N* = 2), and fatigue (*N* = 2), among others. Grade ≥3 adverse events related to riluzole included fatigue (*N* = 1) and abdominal pain (*N* = 1). There were 2 deaths within 30 days of the last dose of study drugs that were attributed to disease progression and unrelated to protocol therapy.

### Tumor response

**Figure 1:**
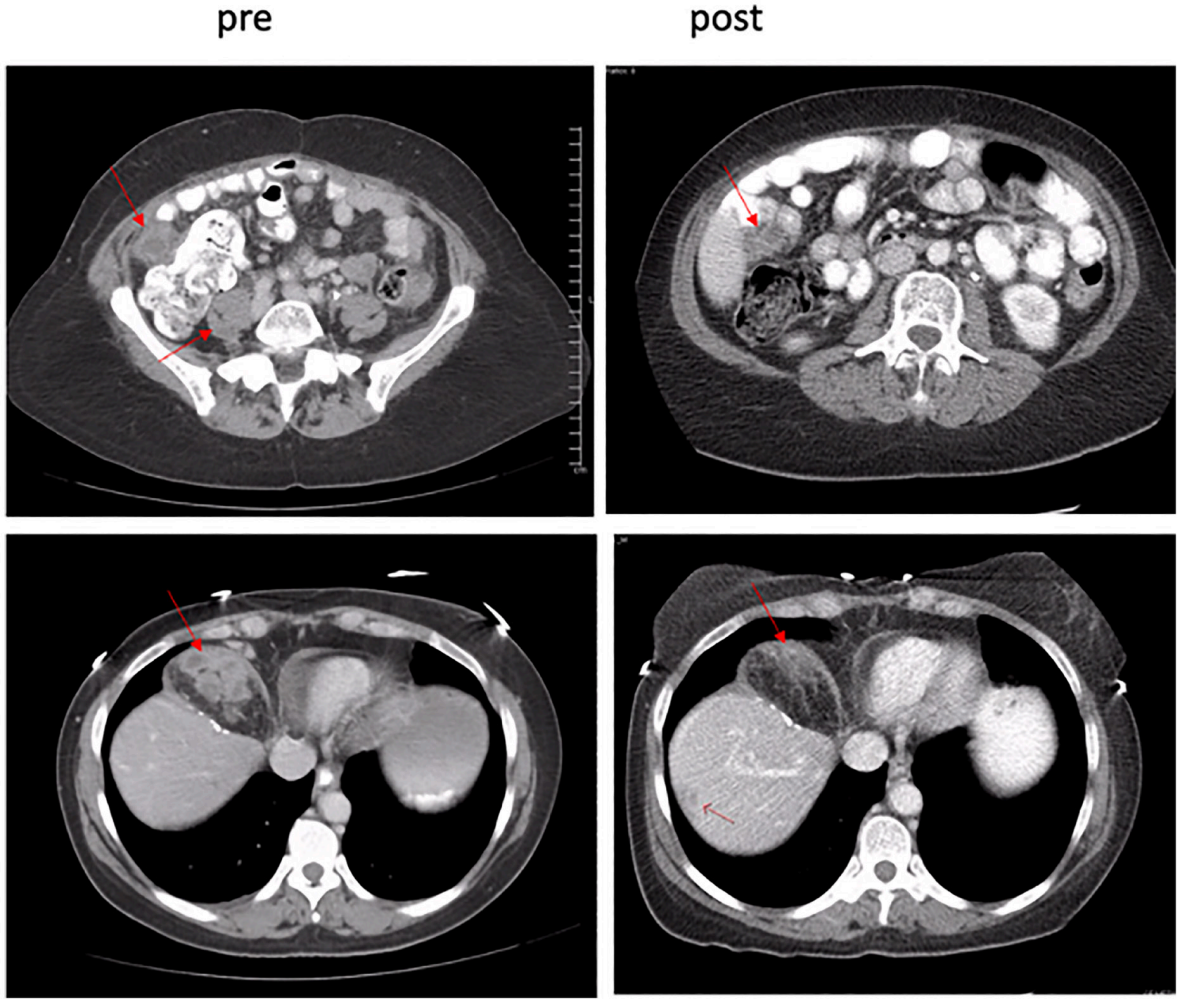
Pre- and post-treatment scans confirmed partial responder. A 39.9% decrease in target lesions was observed after C2D1.

Thirty patients (86%) were evaluable for treatment efficacy and tumor response. Five patients discontinued protocol therapy prior to the first scan and were not evaluable. Two patients discontinued therapy due to adverse events possibly related to study treatment, one patient with grade 3 fatigue possibly related to riluzole or sorafenib, and one patient with grade 4 troponin elevation, grade 4 non-ST segment elevation myocardial infarction (NSTEMI), and grade 3 congestive heart failure in the setting of a prior history of coronary artery disease (CAD). The remaining three patients discontinued therapy for non-treatment related reasons, including 1 patient who discontinued treatment due to a hip fracture that was unrelated to treatment, and 2 patients who were discontinued due to protocol nonadherence. Eleven patients (36%) experienced stable disease. The median time from on study to date of progression was 106 days (range 51–240 days) or 3.5 months (range 1.7–8.0 months). Only one patient had stable disease for over six months, a patient with a metastatic gastrointestinal stromal tumor (GIST) previously treated with imatinib, nilotinib, and sunitinib who was on protocol therapy for 8 months before progression. Eighteen patients (51%) experienced progressive disease. One patient with metastatic pancreatic acinar cell cancer experienced a partial response lasting 264 days (8.8 months) (Figure 1). The median number of months on trial was 3.3 (range 1.4–9.2 months).

### Pharmacokinetics

**Figure 2:**
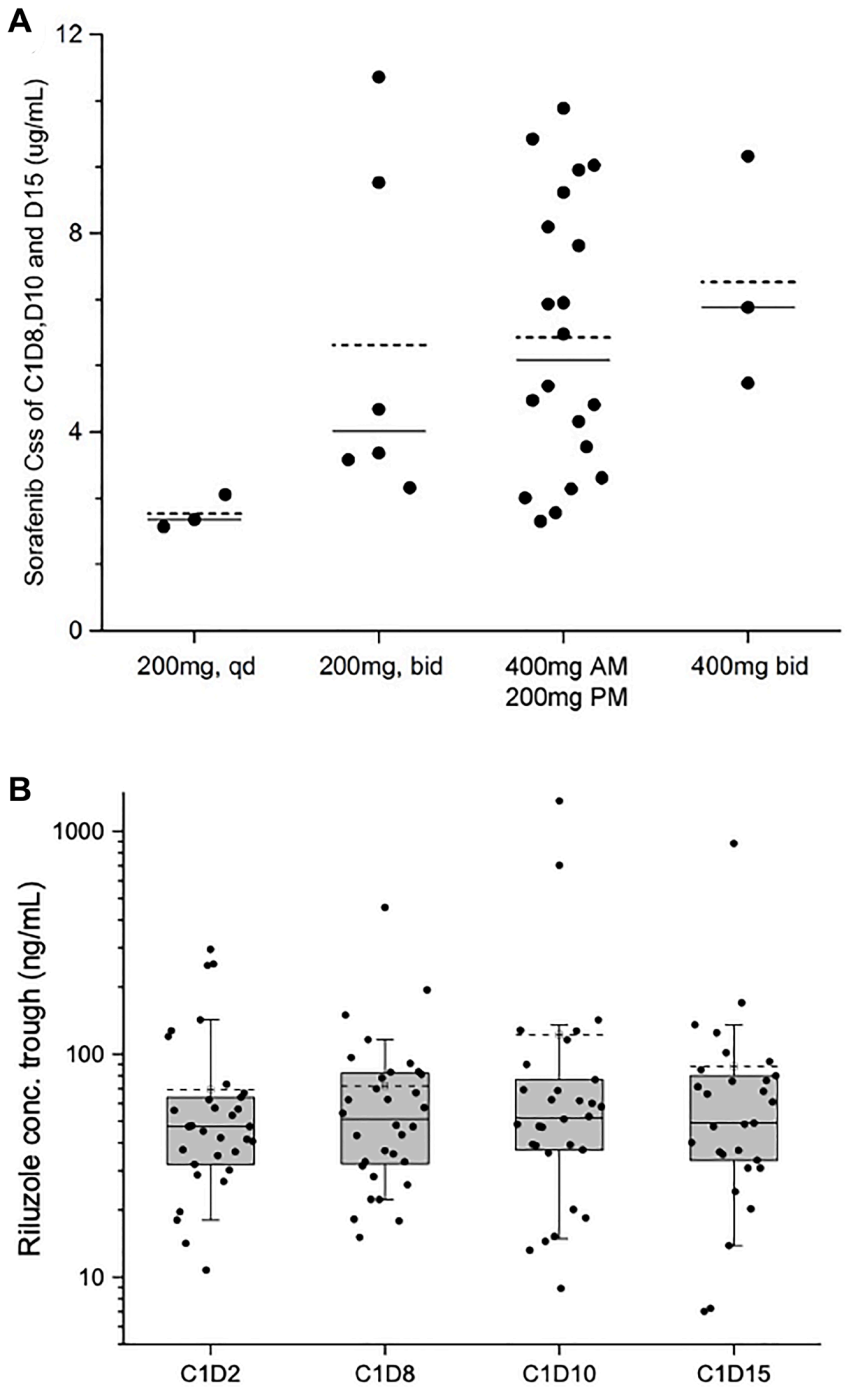
Sorafenib Cmin,ss values at dose escalations and riluzole trough concentrations at different time points. (**A**) Sorafenib average Cmin,ss of C1D8, D10, and D15 vs. different dose level. (–––) median of the data set; (-----) mean of the data set. (**B**) Boxplot of riluzole trough plasma level on C1D2, D8, D10, and D15. (–––) median of the data set; (-----) mean of the data set.

Pharmacokinetic data were available from all 35 patients for both riluzole and sorafenib. The plasma concentration steady state (Cmin,ss) of sorafenib was achieved on C1D8. In this sorafenib/riluzole combination study substantial interpatient variation in sorafenib Cmin,ss was observed at each dose level (Supplementary Table 2). The plasma Cmin,ss levels showed a dose proportional increase with dose escalation from DL 1 to 2 (200 mg qd to 200 mg BID sorafenib), however, there was no proportional increase in Cmin,ss levels with subsequent dose level escalations (Figure 2A). At the MTD, there was substantial accumulation of plasma sorafenib levels with multiple doses as indicated by an accumulation factor of 2.58 (Supplementary Table 3).

Plasma riluzole levels achieved steady state after eight days of continuous dosing and no riluzole accumulation was observed through multiple doses (Figure 2B; Supplementary Table 3). In this sorafenib/riluzole combination study, the plasma Cmin,ss (geometric mean) of riluzole was 55.83 ng/mL (Day 10) and 51.47 ng/mL (Day 15), which were less than the steady state levels in healthy volunteers [22] and in patients with advanced melanoma with multiple doses of riluzole [17]. The riluzole trough levels significantly varied among patients. However, despite inter-patient variability, intra-patient plasma riluzole trough levels were largely consistent from C1D2 to C1D15, suggesting that the disposition of riluzole in the body may be modulated by host specific factors such as absorption from the gut or non-extractable binding to transport proteins and other systemic factors like hepatic metabolism and elimination (Supplementary Table 4) [23].

No significant difference in the population mean of riluzole Cmin,ss was observed in patients treated across dose levels with different doses of sorafenib as analyzed by ANOVA analysis at a significance level of *p* < 0.05. However, at DL4, with sorafenib doses increased to 400 mg BID, a decrease of riluzole Cmin,ss was observed in three patients (geometric mean, 25.98 ng/mL) (Supplementary Table 3). With this relatively lower Cmin,ss of 25.98 ng/mL at DL4, we sought to determine whether higher doses of sorafenib interfere with the hepatic metabolism and bioavailability of riluzole. Since the systemic metabolism of sorafenib is by cytochrome P450 isoform CYP3A4 [24] and riluzole is by CYP1A2 [25], drug-drug interactions were not anticipated. To rule out the potential DDI, the PK data from DL3 were further analyzed in two groups, those who had high sorafenib Cmin,ss (defined as >4 μg/mL) and those with low (defined as <4 μg/mL). Riluzole levels at DL3 were compared with the total median of this study (49.06 ng/mL on Day 15, Supplementary Table 4). The geomean of sorafenib Cmin,ss in Group 1 (“high”) (14 patients, 70% of the cohort) was 7.3 μg/mL (range 4.5–10.5 μg/mL) and of riluzole Cmin,ss was 82.1 ng/mL (range 46–793 ng/mL). The geomean of sorafenib Cmin,ss in Group 2 (“low”) (6 patients, 30%) was 2.8 μg/mL (range 2.2–3.7 μg/mL) with riluzole Cmin,ss at 74.8 ng/mL. The relatively high concentrations of riluzole achieved across both groups imply that drug-drug interactions of significance did not occur, although a larger patient sample size would be necessary to confirm this. These data suggest that the low levels of riluzole observed in DL4 may be due to missed doses due to toxicity, presystemic poor absorption from the gut, and other individual patient factors.

### Correlative studies

**Figure 3:**
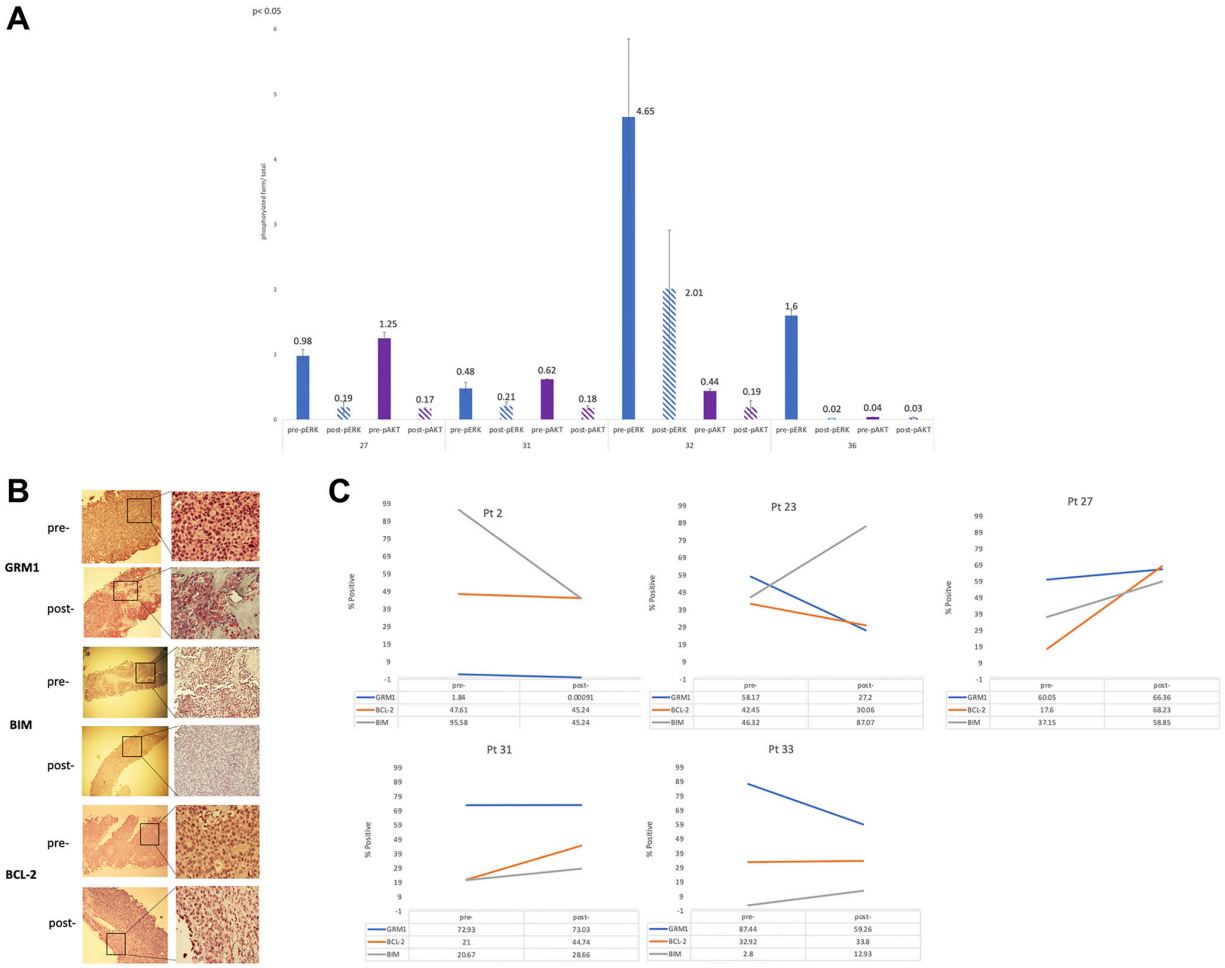
(**A**) Quantification of 4 paired pre- and post-treatment patient samples, protein lysates prepared and subjected to Western immunoblots with pre-pERK, post-pERK, pre-pAKT and post-PAKT. The values of the quantifications were the mean ± STDEV of three independent experiments of the phosphorylated form over total form. Student’s *t*-test was used to calculate statistical significance. ^*^ *p* < 0.05. (**B**) IHC staining pre- and post-treatment tissue of patient 33 whose best response was partial response. Pre- and post-staining for GRM1, BIM and BCL-2 for pt 33 was 87.44% to 59.26%, 2.8% to 12.93%, and 32.92% to 33.8%. Values for GRM1 and BIM were statistically significant (*p* < 0.05). (**C**) Trends in immunohistochemical staining for GRM1, BIM, and BCL-2 for 5 patients.

Pre- and post-treatment tumor tissue was available from 9 patients. Supplemental Table 5 provides an overall summary of the results for the correlative studies performed to date. Pre- and post-treatment tumor samples from 6 patients were evaluated by Western immunoblots for quantification of the intensities of specific bands for pERK, ERK, pAKT and AKT, and pCRAF and CRAF (See Supplementary Figure 1). Statistically significant (*p* < 0.05) changes in levels of pERK and pAKT were seen for 4 of the 6 patients, expressed as a ratio of total ERK and AKT and presented in Figure 3A (See Supplementary Table 5 for all patients). Phospho-forms of ERK and AKT demonstrated statistically significant (*p* < 0.05) post-treatment decreases in pERK and pAKT (Figure 3A) for each of the 4 patients.

An example of IHC staining for GRM1, BIM and BCL-2 in a patient who experienced a partial response and clinical benefit is shown in Figure 3B. Pre- and post-treatment trends in IHC samples for GRM1, BCL-2 and BIM are further presented in Figure 3C. A total of 6 patients had evaluable tissue for IHC analysis of GRM1, 4 of which (patient 23 with sarcoma, patient 33 with acinar cell pancreas, patients 2 and 26 with melanoma) showed statistically significant (*p* < 0.05) decrease in GRM1 expression from pre- to post-treatment tissue (Figure 3C, Supplementary Table 5). Pro- and anti-apoptotic protein expression was measured, with the expectation that a profile of increased pro-apoptotic BIM and decreased anti-apoptotic BCL-2 would be observed. While BIM expression was increased in the majority of post-treatment samples tested (patient 23, sarcoma; 27 and 31, melanoma; and 33, pancreas; *p* < 0.05), consistent with evidence of MAPK blockade, clear trends in BCL-2 expression were not observed. Of note, the one patient who achieved a partial response (pancreatic acinar cell carcinoma with KANK4-RAF1 fusion identified by clinical-grade next generation sequencing) demonstrated the most dramatic change in BIM expression (Figure 3, Supplementary Table 5).

## DISCUSSION

Over-expression of the metabotropic glutamate receptor 1 (GRM1) has been implicated in the facilitation of endothelial cell growth in a number of solid malignancies by way of activation of the MAPK and PI3K/AKT signaling pathways. Riluzole functions as an inhibitor of GRM1 signaling through antagonism of glutamate release, and sorafenib is a multi-kinase inhibitor targeting both the MAPK and PI3K/AKT pathways through the inhibition of RAF1, ARAF and, to a lesser extent BRAF, as well as a set of tyrosine kinases including VEGFR. Our phase I study determined the tolerable dose of this combination and investigated its biologic effects.

The 35 patients enrolled on the study experienced toxicities consistent with known side effect profiles of tyrosine kinase inhibitors including fatigue, lymphopenia, nausea, diarrhea, PPE, and rash. While the most common grade ≥3 toxicity and dose limiting toxicity was rash, only 6 patients experienced dose interruptions, 4 of which required subsequent dose reductions. As a result, the MTD was defined as riluzole 100 mg BID and sorafenib 400 mg qam/200 mg qpm.

Plasma pharmacokinetic studies for this trial were planned to determine whether significant drug-drug interactions occurred with this regimen. The large inter-individual variability in plasma riluzole levels (35% to 175% CV in the geometric mean among individuals in various sorafenib dose cohorts) observed in this study limits our conclusions. High variability in plasma riluzole levels has been reported in patients with ALS [26, 27], and has been attributed to pre-systemic metabolism and polymorphic hepatic cytochrome P450 metabolism with oral administration of riluzole [28]. Indeed, this interpatient variability, as well as overall lower bioavailability, is a known difficulty in the administration of riluzole for neurologic diseases. These challenges have led to the search for next generation antagonists of glutamate signaling such as troriluzole (BHV-4157), which is actively absorbed in the gut via the peptide transporter PepT1, and thus is not subject to a negative food effect, bypasses first-pass metabolism, reduces riluzole burden on the liver, and can be administered once daily. This compound has also entered cancer clinical trials in combination with immune checkpoint blockade (NCT03229278).

The PK parameters of sorafenib such as plasma Cmin,ss, accumulation of sorafenib in the plasma after multiple doses, and high interpatient variability in the current combination study were similar to those of pharmacokinetic reports in previous phase I clinical trials of single agent sorafenib [29, 30]. Sorafenib, like many anticancer drugs, often shows a narrow therapeutic index and high inter-patient variability, making dose adaptation an important part of optimizing the efficacy and tolerability of this agent. Thus, accurate measurement of toxicity in small samples sizes, such as those used in phase I trials, can be affected by this inter-patient variability. Our pharmacokinetic analysis, limited by few patients at all dose levels, did not reveal evidence of drug-drug interaction between riluzole and sorafenib. While the riluzole Cmin,ss in plasma in combination with sorafenib was, for the most part, comparable to the steady state levels in healthy volunteers [22], the decrease in Cmin,ss observed in three patients administered sorafenib 400 mg BID (geomean, 25.98 ng/mL; *n* = 3) was significantly lower than the trough levels (geomean 78.4 ng/mL; *n* = 9) on Day 10 of riluzole single agent treatment (100 mg BID) in advanced melanoma patients [17]. Thus, while it is reasonable to speculate that DDI may exist based on high levels of plasma protein binding for both drugs, as well as the fact that increased riluzole hepatic clearance may be a consequence of an increase in its plasma free fraction due to displacement by sorafenib, the presence of DDI cannot be confirmed or ruled out given the small number of patients enrolled in this study.

Within the small subset of patients who had both pre- and post-treatment tissue available for correlative testing, consistent and statistically significant decreases in phospho-forms of both ERK and AKT in a majority of patients suggested target engagement by the combination of riluzole and sorafenib, with accompanying decreases in GRM1 expression and increases in the pro-apoptotic protein BIM which suggest efficacy. The lack of consistent decreases in BCL-2 protein expression may reflect the dynamic nature of the apoptotic response. While these studies are hypothesis-generating due to small sample size, the results support our hypothesis that MAPK and PI3K signaling pathways are affected by the riluzole and sorafenib treatment regimen.

Although the majority of patients had progressive disease with this combination (51%), one patient with pancreatic acinar cell carcinoma with metastases to the liver and mesenteric lymph nodes whose tumor harbored a KANK4-RAF1 fusion identified by next generation sequencing achieved a partial response that encouragingly lasted for over 8 months. This patient was heavily pretreated with multiple (>5) prior lines of chemotherapy as well as chemoembolization of liver metastases, none of which resulted in a clinical response. In addition to a radiologic and clinical response, this patient displayed statistically significant decreases in GRM1, AKT, and pro-apoptotic protein BIM in analyses of paired tumor tissue, supporting target engagement and activity. The clinical response sustained over several months and the almost 4-fold increase in BIM levels post-treatment in this patient suggests further investigation of this combination in a pre-specified subset of patients may be warranted.

Multiple gene fusions involving RAF1 have been implicated in the pathogenesis of solid organ tumors including pancreatic acinar cell carcinomas [31–37], and it has been suggested that these fusions are associated with advanced pathologic features [32]. Of note, sorafenib is a potent inhibitor of RAF1 with IC50 of ~6 nM [38]. Though it is possible that the response seen in the patient with the RAF1 fusion was due to sorafenib alone, some contribution of riluzole cannot be ruled out. Glutamate signaling and metabolism has been implicated in growth and survival of many cancer types and may be a broader target than in melanoma alone [8]. Further analyses of sensitivity of cell and animal models of RAF1-fusion driven cancers to sorafenib, riluzole and the combination are in progress and may be helpful in guiding future clinical trials.

In conclusion, the combination of riluzole and sorafenib was not recommended for further broad empiric study, however, the partial response seen in a patient with metastatic pancreatic acinar cell carcinoma harboring a KANK-RAF1 fusion, accompanied by a decrease in GRM1 expression in correlative studies suggests that molecularly defined subsets of cancers may be sensitive to this combination. Alternatively, combination studies of sorafenib of riluzole with next generation riluzole prodrug candidates can be entertained. Further studies in a pre-selected patient population harboring RAF1 aberrations, and including ARAF aberrations and non-canonical BRAF mutations are under consideration.

## MATERIALS AND METHODS

### Study design and patients

This was an open label phase I dose escalation trial conducted at Rutgers Cancer Institute of New Jersey (NCT01303341, NCI-CTEP #8850). Eligible patients were ≥18 years with solid tumors refractory to standard therapy or for whom no standard therapy existed, with a planned expansion cohort in patients with mandatory paired tumor biopsies for correlative studies at the recommended safe dose of the combination. There were no restrictions on the number of prior therapies; however, patients enrolled into the expansion cohort were excluded if they had prior therapy with riluzole or sorafenib. Other key inclusion criteria included an Eastern Cooperative Oncology Group (ECOG) performance status ≤2, clinically stable brain lesions for at least 4 weeks, adequate hematological function (ANC ≥1,500/μL, platelets ≥100,000 μL), adequate hepatic function (total bilirubin ≤1.5 X institutional ULM, AST(SGOT)/SLT(SGPT) ≤2.5 X institutional ULN), INR ≤1.5 institutional ULN, and adequate renal function (creatinine ≤2X ULN). The study protocol was approved by the Rutgers Robert Wood Johnson Medical School Institutional Review Board (NCT01303341) and written informed consent was obtained from all patients.

### Primary and secondary objectives

The primary objective was to determine the dose limiting toxicities (DLTs) and maximum tolerated dose (MTD) of sorafenib in combination with riluzole. Secondary objectives included characterizing the pharmacokinetic profile, and examining the correlation between clinical or radiographic response with GRM1 expression, MAPK/PI3K/AKT pathway signaling components, and expression of apoptotic protein family members including BCL-2 and BIM.

### Treatment

This phase I trial utilized a standard 3+3 dose escalation schema. Four dose levels were evaluated with a cycle defined as a period of 28 days. The dose of riluzole was kept constant at 100 mg PO twice a day (BID). While a dose-escalation phase I trial was not performed with riluzole in cancer patients, we chose the 100 mg BID dose based on the evidence of biologic activity and overall safety in our phase 0 and Phase II trials at 100 mg BID [16, 17], as well as its use in clinical practice with ALS patients as the highest tolerable dose. The dose of sorafenib (NCI-supplied agent, NSC724774) was escalated from 200 mg PO daily (dose level 1) to 200 mg BID (dose level 2), 400 mg AM/200 mg PM (dose level 3), and 400 mg BID (dose level 4). The FDA approved recommended dose of sorafenib as monotherapy is 800 mg per day (400 mg orally BID). All patients were instructed to take riluzole and sorafenib together at least one hour before or two hours after a meal to avoid a food related decrease in bioavailability.

### Rationale for dose selection

A fixed dose of riluzole, 100 mg BID, was selected for this study as we previously used the same fixed dose in a phase 0 trial in patients with stage III and IV resectable melanoma and a phase II trial in melanoma [16]. Since riluzole and sorafenib have not been combined previously in human subjects, a lower starting dose of sorafenib (200 mg qd) was used.

### Safety evaluations

A cycle was defined as a period of 4 weeks or 28 days. Safety assessments including complete blood counts, serum chemistries, liver enzymes and toxicity evaluations were completed at baseline, day 1 and 15 of cycle 1, and then day 1 of every cycle thereafter. Toxicities were evaluated and graded using the CTEP NCI Common Terminology Criteria for Adverse Events (CTCAE), version 4.0. All patients were evaluable for toxicity from the time of their first treatment of riluzole and sorafenib.

### Tumor response evaluations

Disease response assessments by imaging were performed after every 2 cycles of treatment (approximately every 8 weeks). Response and progression were evaluated using the Response Evaluation Criteria in Solid Tumors (RECIST) version 1.1 [39]. All patients who had measurable disease at baseline, had received at least one cycle of therapy, and had their disease re-evaluated were considerable evaluable for response.

### Pharmacokinetic analyses

Based on previous reports that after multiple continuous doses of riluzole [17, 22, 23] and of sorafenib [40], steady state plasma levels were reached after seven days, blood samples (8 mL) were collected from all patients prior to drug administration on day 2, 8, 10, and 15 of the first cycle in heparinized vacutainer tubes (Beckton Dickinson, Franklin Lakes, NJ). Following centrifugation for 10 min at 1,500 × g at 4°C within 30 min of blood collection, the plasma was aliquoted and stored at −80°C. Concentrations of sorafenib and riluzole in plasma were analyzed using a validated liquid chromatography-tandem mass spectrometry method (LC-MS/MS) as described previously [41, 42], with the lower limits of quantitation at 31 ng/mL and 1.9 ng/mL, respectively.

### Correlative studies

To determine the effects of the combination of riluzole and sorafenib on pathway signaling components and confirm target engagement, paired tumor samples were collected at baseline (pre-treatment) and after 28 days at cycle 2 day 1. Paired tumor biopsies were optional in the dose escalation portion of the study, but required for patients in the expansion cohort. Tumor samples were split into two blocks. The first block was flash frozen and stored in a −80^°C^ freezer until processed for Western blotting. Approximately 500 mg of tumor tissue was homogenized and lysed on ice for 45 minutes in RIPA buffer (10 mM sodium phosphate, pH 7.2, 1% nonidet P-40, 1% sodium deoxycholate, 0.1% SDS, 150 mM NaCl, 2 mM EDTA) supplemented with fresh 1% aprotinin, 1 mM phenylmethylsulfonyl fluoride, and 50 μg/ml leupeptin, and centrifuged at 14,000 × g at 4°C for 10 minutes. Proteins were resolved by 10% SDS-PAGE and transferred to nitrocellulose membranes. The blots were incubated in blocking solution consisting of 5% non-fat milk in TBS-T (0.1% Tween-20) for 1 hour at 25^o^C, then immunoblotted with polyclonal antibody specific for the target protein. Tissue samples were examined for expression of phosphorylated and total ERK, AKT, and CRAF.

In order to determine the degree of GRM1 expression and apoptosis resulting from combination therapy, the second part of the tumor samples was processed for immunohistochemical (IHC) analysis of expression of GRM1, BCL-2, and BIM. Paraffin-embedded tissue was paraffinized and endogenous peroxidase activity was blocked with dilute H_2_O_2_. Sections were blocked with 5% bovine serum albumin (BSA) for 15 minutes, washed with PBS, and then incubated with the optimized polyclonal antibody at the optimal concentration in 1% BSA in PBS overnight. Sections were washed twice with cold PBS and incubated with Biotinylated secondary antibody (BD PharMingen, San Diego, CA, USA) in 1% BSA/PBS at 1:400 dilution. Color was developed with streptavidin-peroxidase (VectaStain, ABC Kit, Vector Laboratories, Burlingame, CA, USA). Stained slides were scored by Histowiz (Brooklyn, NY, USA) as the number of cells with 0 (none), 1+ (minimal), 2+ (moderate), or 3+ (high), by the IHC scoring methods as described for Her-2/neu [43, 44]. The percent calculated was based on cells with at least 1+ positive stain over the total number of cells scored.

### Statistical analyses

For the primary objective of defining a safe dose of sorafenib in combination with riluzole, a standard 3+3 approach was used, with 4 dose levels used as described above. The MTD was defined as the first dose level at which exactly 2/6 patients experienced a DLT, or at which 1/6 experienced a DLT and (due to de-escalation) at least 2/3 or 3/6 patients treated with the next higher dose level had a DLT. Once the MTD was identified, enrollment of 12 patients at the recommended safe dose was planned. Patients were monitored for DLTs during the first cycle (28 days) of treatment. A DLT was defined as any significant grade 3 or 4 non-hematological toxicity, grade 4 neutropenia that persisted >5 days or was associated with fever, or grade 4 anemia or thrombocytopenia that occurred during the first cycle of treatment and was attributed to the study treatment.

Descriptive statistics were used for the analysis of study parameters, endpoints, and data including patient characteristics and toxicity data. In analyzing the significance of the effect of increasing sorafenib doses on riluzole PK, ANOVA analysis at 0.05 level was used. Student *t*-test was used to calculate statistical significance (*p* < 0.05) of difference in the levels of phosphorylated forms of ERK, AKT, and CRAF in pre- vs. post-treatment patient tumor samples. Pre- and post-treatment levels of GRM1, BCL-2, and BIM were compared using appropriate parametric and nonparametric methods.

## SUPPLEMENTARY MATERIALS



## References

[1] Arcella A , Carpinelli G , Battaglia G , D’Onofrio M , Santoro F , Ngomba RT , Bruno V , Casolini P , Giangaspero F , Nicoletti F . Pharmacological blockade of group II metabotropic glutamate receptors reduces the growth of glioma cells *in vivo* . Neuro Oncol. 2005; 7:236–45. 10.1215/S1152851704000961. 16053698 PMC1871912

[2] Aronica E , Gorter JA , Ijlst-Keizers H , Rozemuller AJ , Yankaya B , Leenstra S , Troost D . Expression and functional role of mGluR3 and mGluR5 in human astrocytes and glioma cells: opposite regulation of glutamate transporter proteins. Eur J Neurosci. 2003; 17:2106–18. 10.1046/j.1460-9568.2003.02657.x. 12786977

[3] D’Onofrio M , Arcella A , Bruno V , Ngomba RT , Battaglia G , Lombari V , Ragona G , Calogero A , Nicoletti F . Pharmacological blockade of mGlu2/3 metabotropic glutamate receptors reduces cell proliferation in cultured human glioma cells. J Neurochem. 2003; 84:1288–95. 10.1046/j.1471-4159.2003.01633.x. 12614329

[4] Brocke KS , Staufner C , Luksch H , Geiger KD , Stepulak A , Marzahn J , Schackert G , Temme A , Ikonomidou C . Glutamate receptors in pediatric tumors of the central nervous system. Cancer Biol Ther. 2010; 9:455–68. 10.4161/cbt.9.6.10898. 20061814

[5] Speyer CL , Smith JS , Banda M , DeVries JA , Mekani T , Gorski DH . Metabotropic glutamate receptor-1: a potential therapeutic target for the treatment of breast cancer. Breast Cancer Res Treat. 2012; 132:565–73. 10.1007/s10549-011-1624-x. 21681448 PMC3898178

[6] Koochekpour S , Majumdar S , Azabdaftari G , Attwood K , Scioneaux R , Subramani D , Manhardt C , Lorusso GD , Willard SS , Thompson H , Shourideh M , Rezaei K , Sartor O , et al. Serum glutamate levels correlate with Gleason score and glutamate blockade decreases proliferation, migration, and invasion and induces apoptosis in prostate cancer cells. Clin Cancer Res. 2012; 18:5888–901. 10.1158/1078-0432.CCR-12-1308. 23072969 PMC3492499

[7] Namkoong J , Shin SS , Lee HJ , Marín YE , Wall BA , Goydos JS , Chen S . Metabotropic glutamate receptor 1 and glutamate signaling in human melanoma. Cancer Res. 2007; 67:2298–305. 10.1158/0008-5472.CAN-06-3665. 17332361

[8] Stepulak A , Luksch H , Gebhardt C , Uckermann O , Marzahn J , Sifringer M , Rzeski W , Staufner C , Brocke KS , Turski L , Ikonomidou C . Expression of glutamate receptor subunits in human cancers. Histochem Cell Biol. 2009; 132:435–45. 10.1007/s00418-009-0613-1. 19526364

[9] Pollock PM , Cohen-Solal K , Sood R , Namkoong J , Martino JJ , Koganti A , Zhu H , Robbins C , Makalowska I , Shin SS , Marin Y , Roberts KG , Yudt LM , et al. Melanoma mouse model implicates metabotropic glutamate signaling in melanocytic neoplasia. Nat Genet. 2003; 34:108–12. 10.1038/ng1148. 12704387

[10] Wen Y , Li J , Koo J , Shin SS , Lin Y , Jeong BS , Mehnert JM , Chen S , Cohen-Sola KA , Goydos JS . Activation of the glutamate receptor GRM1 enhances angiogenic signaling to drive melanoma progression. Cancer Res. 2014; 74:2499–509. 10.1158/0008-5472.CAN-13-1531. 24491800 PMC4008638

[11] Pópulo H , Lopes JM , Soares P . The mTOR signalling pathway in human cancer. Int J Mol Sci. 2012; 13:1886–918. 10.3390/ijms13021886. 22408430 PMC3291999

[12] Steelman LS , Chappell WH , Abrams SL , Kempf RC , Long J , Laidler P , Mijatovic S , Maksimovic-Ivanic D , Stivala F , Mazzarino MC , Donia M , Fagone P , Malaponte G , et al. Roles of the Raf/MEK/ERK and PI3K/PTEN/Akt/mTOR pathways in controlling growth and sensitivity to therapy-implications for cancer and aging. Aging (Albany NY). 2011; 3:192–222. 10.18632/aging.100296. 21422497 PMC3091517

[13] Shah R , Singh SJ , Eddy K , Filipp FV , Chen S . Concurrent Targeting of Glutaminolysis and Metabotropic Glutamate Receptor 1 (GRM1) Reduces Glutamate Bioavailability in GRM1^+^ Melanoma. Cancer Res. 2019; 79:1799–809. 10.1158/0008-5472.CAN-18-1500. 30987979 PMC6469683

[14] Le MN , Chan JL , Rosenberg SA , Nabatian AS , Merrigan KT , Cohen-Solal KA , Goydos JS . The glutamate release inhibitor Riluzole decreases migration, invasion, and proliferation of melanoma cells. J Invest Dermatol. 2010; 130:2240–49. 10.1038/jid.2010.126. 20505744 PMC4004181

[15] Wangari-Talbot J , Wall BA , Goydos JS , Chen S . Functional effects of GRM1 suppression in human melanoma cells. Mol Cancer Res. 2012; 10:1440–50. 10.1158/1541-7786.MCR-12-0158. 22798429 PMC3501593

[16] Yip D , Le MN , Chan JL , Lee JH , Mehnert JA , Yudd A , Kempf J , Shih WJ , Chen S , Goydos JS . A phase 0 trial of riluzole in patients with resectable stage III and IV melanoma. Clin Cancer Res. 2009; 15:3896–902. 10.1158/1078-0432.CCR-08-3303. 19458050 PMC2812866

[17] Mehnert JM , Silk AW , Lee JH , Dudek L , Jeong BS , Li J , Schenkel JM , Sadimin E , Kane M , Lin H , Shih WJ , Zloza A , Chen S , Goydos JS . A phase II trial of riluzole, an antagonist of metabotropic glutamate receptor 1 (GRM1) signaling, in patients with advanced melanoma. Pigment Cell Melanoma Res. 2018; 31:534–40. 10.1111/pcmr.12694. 29453787 PMC6013351

[18] Palmieri G , Ombra M , Colombino M , Casula M , Sini M , Manca A , Paliogiannis P , Ascierto PA , Cossu A . Multiple Molecular Pathways in Melanomagenesis: Characterization of Therapeutic Targets. Front Oncol. 2015; 5:183. 10.3389/fonc.2015.00183. 26322273 PMC4530319

[19] Hawryluk EB , Tsao H . Melanoma: clinical features and genomic insights. Cold Spring Harb Perspect Med. 2014; 4:a015388. 10.1101/cshperspect.a015388. 25183853 PMC4143108

[20] Rosenberg SA , Niglio SA , Salehomoum N , Chan JL , Jeong BS , Wen Y , Li J , Fukui J , Chen S , Shin SS , Goydos JS . Targeting Glutamatergic Signaling and the PI3 Kinase Pathway to Halt Melanoma Progression. Transl Oncol. 2015; 8:1–9. 10.1016/j.tranon.2014.11.001. 25749171 PMC4350641

[21] Lee HJ , Wall BA , Wangari-Talbot J , Shin SS , Rosenberg S , Chan JL , Namkoong J , Goydos JS , Chen S . Glutamatergic pathway targeting in melanoma: single-agent and combinatorial therapies. Clin Cancer Res. 2011; 17:7080–92. 10.1158/1078-0432.CCR-11-0098. 21844014 PMC3218300

[22] Le Liboux A , Lefebvre P , Le Roux Y , Truffinet P , Aubeneau M , Kirkesseli S , Montay G . Single- and multiple-dose pharmacokinetics of riluzole in white subjects. J Clin Pharmacol. 1997; 37:820–27. 10.1002/j.1552-4604.1997.tb05630.x. 9549636

[23] Grant P , Farmer C , Song J , Kish T , Swedo S . Riluzole Serum Concentration in Pediatric Patients Treated for Obsessive-Compulsive Disorder. J Clin Psychopharmacol. 2017; 37:713–16. 10.1097/JCP.0000000000000797. 29045303 PMC5679444

[24] Boudou-Rouquette P , Narjoz C , Golmard JL , Thomas-Schoemann A , Mir O , Taieb F , Durand JP , Coriat R , Dauphin A , Vidal M , Tod M , Loriot MA , Goldwasser F , Blanchet B . Early sorafenib-induced toxicity is associated with drug exposure and UGTIA9 genetic polymorphism in patients with solid tumors: a preliminary study. PLoS One. 2012; 7:e42875. 10.1371/journal.pone.0042875. 22912756 PMC3418266

[25] van Kan HJ , Groeneveld GJ , Kalmijn S , Spieksma M , van den Berg LH , Guchelaar HJ . Association between CYP1A2 activity and riluzole clearance in patients with amyotrophic lateral sclerosis. Br J Clin Pharmacol. 2005; 59:310–13. 10.1111/j.1365-2125.2004.02233.x. 15752377 PMC1884790

[26] Groeneveld GJ , van Kan HJ , Lie-A-Huen L , Guchelaar HJ , van den Berg LH . An association study of riluzole serum concentration and survival and disease progression in patients with ALS. Clin Pharmacol Ther. 2008; 83:718–22. 10.1038/sj.clpt.6100382. 17898704

[27] Groeneveld GJ , van Kan HJ , Toraño JS , Veldink JH , Guchelaar HJ , Wokke JH , van den Berg LH . Inter- and intraindividual variability of riluzole serum concentrations in patients with ALS. J Neurol Sci. 2001; 191:121–25. 10.1016/s0022-510x(01)00613-x. 11677002

[28] Dash RP , Babu RJ , Srinivas NR . Two Decades-Long Journey from Riluzole to Edaravone: Revisiting the Clinical Pharmacokinetics of the Only Two Amyotrophic Lateral Sclerosis Therapeutics. Clin Pharmacokinet. 2018; 57:1385–98. 10.1007/s40262-018-0655-4. 29682695

[29] Jain L , Woo S , Gardner ER , Dahut WL , Kohn EC , Kummar S , Mould DR , Giaccone G , Yarchoan R , Venitz J , Figg WD . Population pharmacokinetic analysis of sorafenib in patients with solid tumours. Br J Clin Pharmacol. 2011; 72:294–305. 10.1111/j.1365-2125.2011.03963.x. 21392074 PMC3162659

[30] Strumberg D , Clark JW , Awada A , Moore MJ , Richly H , Hendlisz A , Hirte HW , Eder JP , Lenz HJ , Schwartz B . Safety, pharmacokinetics, and preliminary antitumor activity of sorafenib: a review of four phase I trials in patients with advanced refractory solid tumors. Oncologist. 2007; 12:426–37. 10.1634/theoncologist.12-4-426. 17470685

[31] Giacomini CP , Sun S , Varma S , Shain AH , Giacomini MM , Balagtas J , Sweeney RT , Lai E , Del Vecchio CA , Forster AD , Clarke N , Montgomery KD , Zhu S , et al. Breakpoint analysis of transcriptional and genomic profiles uncovers novel gene fusions spanning multiple human cancer types. PLoS Genet. 2013; 9:e1003464. 10.1371/journal.pgen.1003464. 23637631 PMC3636093

[32] Palanisamy N , Ateeq B , Kalyana-Sundaram S , Pflueger D , Ramnarayanan K , Shankar S , Han B , Cao Q , Cao X , Suleman K , Kumar-Sinha C , Dhanasekaran SM , Chen YB , et al. Rearrangements of the RAF kinase pathway in prostate cancer, gastric cancer and melanoma. Nat Med. 2010; 16:793–98. 10.1038/nm.2166. 20526349 PMC2903732

[33] Kumar-Sinha C , Kalyana-Sundaram S , Chinnaiyan AM . Landscape of gene fusions in epithelial cancers: seq and ye shall find. Genome Med. 2015; 7:129. 10.1186/s13073-015-0252-1. 26684754 PMC4683719

[34] Chmielecki J , Hutchinson KE , Frampton GM , Chalmers ZR , Johnson A , Shi C , Elvin J , Ali SM , Ross JS , Basturk O , Balasubramanian S , Lipson D , Yelensky R , et al. Comprehensive genomic profiling of pancreatic acinar cell carcinomas identifies recurrent RAF fusions and frequent inactivation of DNA repair genes. Cancer Discov. 2014; 4:1398–405. 10.1158/2159-8290.CD-14-0617. 25266736

[35] Jones DT , Kocialkowski S , Liu L , Pearson DM , Ichimura K , Collins VP . Oncogenic RAF1 rearrangement and a novel BRAF mutation as alternatives to KIAA1549:BRAF fusion in activating the MAPK pathway in pilocytic astrocytoma. Oncogene. 2009; 28:2119–23. 10.1038/onc.2009.73. 19363522 PMC2685777

[36] Shimizu K , Nakatsu Y , Nomoto S , Sekiguchi M . Structure of the activated c-raf-1 gene from human stomach cancer. Princess Takamatsu Symp. 1986; 17:85–91. 2843497

[37] Yde CW , Sehested A , Mateu-Regué À , Østrup O , Scheie D , Nysom K , Nielsen FC , Rossing M . A new NFIA:RAF1 fusion activating the MAPK pathway in pilocytic astrocytoma. Cancer Genet. 2016; 209:440–44. 10.1016/j.cancergen.2016.09.002. 27810072

[38] Wilhelm SM , Carter C , Tang L , Wilkie D , McNabola A , Rong H , Chen C , Zhang X , Vincent P , McHugh M , Cao Y , Shujath J , Gawlak S , et al. BAY 43-9006 exhibits broad spectrum oral antitumor activity and targets the RAF/MEK/ERK pathway and receptor tyrosine kinases involved in tumor progression and angiogenesis. Cancer Res. 2004; 64:7099–109. 10.1158/0008-5472.CAN-04-1443. 15466206

[39] Eisenhauer EA , Therasse P , Bogaerts J , Schwartz LH , Sargent D , Ford R , Dancey J , Arbuck S , Gwyther S , Mooney M , Rubinstein L , Shankar L , Dodd L , et al. New response evaluation criteria in solid tumours: revised RECIST guideline (version 1.1). Eur J Cancer. 2009; 45:228–47. 10.1016/j.ejca.2008.10.026. 19097774

[40] Awada A , Hendlisz A , Gil T , Bartholomeus S , Mano M , de Valeriola D , Strumberg D , Brendel E , Haase CG , Schwartz B , Piccart M . Phase I safety and pharmacokinetics of BAY 43-9006 administered for 21 days on/7 days off in patients with advanced, refractory solid tumours. Br J Cancer. 2005; 92:1855–61. 10.1038/sj.bjc.6602584. 15870716 PMC2361774

[41] Le Liboux A , Cachia JP , Kirkesseli S , Gautier JY , Guimart C , Montay G , Peeters PA , Groen E , Jonkman JH , Wemer J . A comparison of the pharmacokinetics and tolerability of riluzole after repeat dose administration in healthy elderly and young volunteers. J Clin Pharmacol. 1999; 39:480–86. 10234595

[42] Haouala A , Zanolari B , Rochat B , Montemurro M , Zaman K , Duchosal MA , Ris HB , Leyvraz S , Widmer N , Decosterd LA . Therapeutic Drug Monitoring of the new targeted anticancer agents imatinib, nilotinib, dasatinib, sunitinib, sorafenib and lapatinib by LC tandem mass spectrometry. J Chromatogr B Analyt Technol Biomed Life Sci. 2009; 877:1982–96. 10.1016/j.jchromb.2009.04.045. 19505856

[43] Mitchell MS , Press MF . The role of immunohistochemistry and fluorescence *in situ* hybridization for HER2/neu in assessing the prognosis of breast cancer. Semin Oncol. 1999; 26:108–16. 10482202

[44] Press MF , Slamon DJ , Flom KJ , Park J , Zhou JY , Bernstein L . Evaluation of HER-2/neu gene amplification and overexpression: comparison of frequently used assay methods in a molecularly characterized cohort of breast cancer specimens. J Clin Oncol. 2002; 20:3095–105. 10.1200/JCO.2002.09.094. 12118023

