# Supplementary material for: A phase I trial of riluzole and sorafenib in patients with advanced solid tumors: CTEP #8850
Source: Oncotarget. 2023 Apr 10;14:302–15. doi: 10.18632/oncotarget.28403 (PMC10085060; doi:10.18632/oncotarget.28403)
Supplement: Supplementary file 1 [file oncotarget-14-28403-s001.pdf]

A phase I trial of riluzole and sorafenib in patients with advanced solid tumors: CTEP #8850

SUPPLEMENTARY MATERIALS

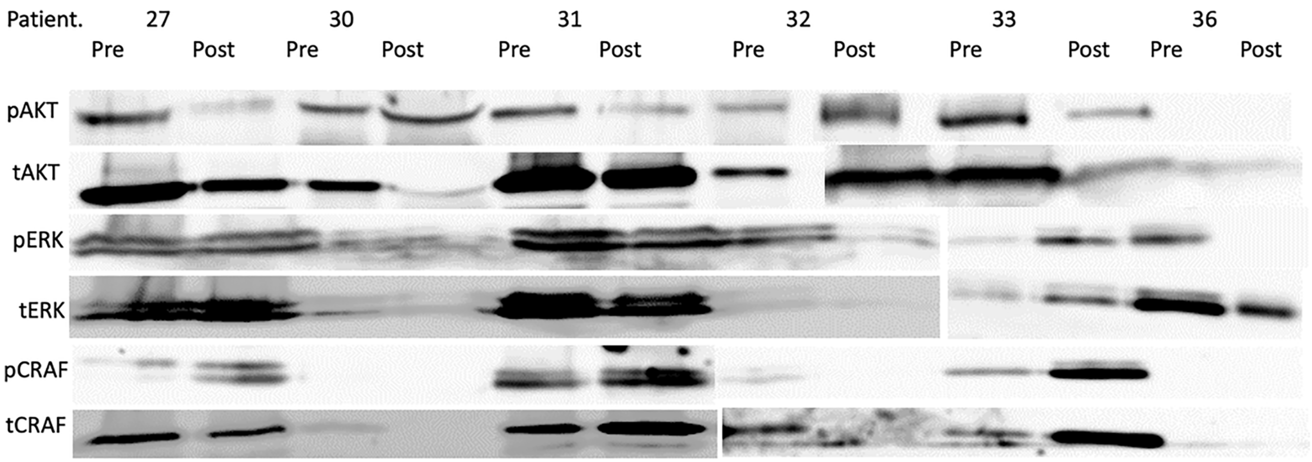

Supplementary Figure 1: Western blots for AKT, ERK, and CRAF for 6 patients.

Supplementary Table 1: Drug related adverse events

Adverse event	Attribution		
	Both S + R	S (only)	R (only)
Fatigue	13 (1)	13 (4)	2 (1)
Nausea	9	2	0
Diarrhea	7	8	0
Anorexia	6 (1)	6	0
Maculopapular Rash	5 (1)	11 (4)	0
Vomiting	4	1	0
Lymphocyte Count Decrease	3 (1)	10 (6)	0
Alanine Aminotransferase Increase	3	1	1
Aspartate Aminotransferase Increase	3	3	2
Headache	3	0	0
Neutrophil count decrease	2 (1)	0	0
Blood Bilirubin Increase	2	2	1
Dysgeusia	2	1	0
Dyspepsia	2	1	0
Flatulence	2	0	0
White Blood Cell Decreased	2	2 (2)	0
Acute Kidney Injury	1	0	0
Alkaline Phosphatase Increase	1	2 (2)	1
Alopecia	1	0	0
Arthralgia	1	1 (1)	1
Blurred vision	1	0	1
Dizziness	1	0	3
Dry skin	1	4	0
Dyspnea	1	0	1
Edema (limbs)	1	1	1
Fever	1	0	0
Gastroparesis	1	0	1
Hoarseness	1	0	0
Hyperglycemia	1	4	0
Hyperkalemia	1	3	0
Hypertension	1	6 (1)	0
Hypocalcemia	1	2	0
Hypokalemia	1	1 (1)	0
Hyponatremia	1	7 (2)	0
Hypophosphatemia	1	11 (4)	0
Lipase Increase	1	5 (2)	0
Myalgia	1	0	0
Palmar-plantar erythrodysesthesia syndrome	1	11 (1)	0
Peripheral Sense Neuropathy	1	1	1
Platelet Count Decrease	1	3	0
Voice Alteration	1	0	0
Acute Coronary Syndrome	0	1	0
Anemia	0	8 (1)	0

Bullous dermatitis	0	1	0
Cardiac troponin I increased	0	1 (1)	0
Dry Mouth	0	1	0
Fecal Incontinence	0	1	0
Gastrointestinal Fistula	0	1	0
Congestive Heart Failure	0	1 (1)	0
Hypercalcemia	0	1	0
Hypoalbuminemia	0	3	0
Hypomagnesemia	0	2	1
Hypotension	0	1	0
Hypothyroidism	0	1	0
Mucosal infection	0	1	0
Mucositis oral	0	5	0
Muscle weakness	0	1	0
Myocardial infarction	0	1 (1)	0
Oral pain	0	1	0
Pruritus	0	4 (1)	0
Increased blood urea nitrogen	0	1	0
Serum amylase increased	0	3	0
Urinary Tract Infection	0	1	0
Weight Loss	0	2	2
Wound complication	0	1 (1)	0
Would dehiscence	0	1	0
Abdominal Pain	0	0	1 (1)
Ataxia	0	0	2
Cough	0	0	1
Depression	0	0	1
Dysarthria	0	0	1
Insomnia	0	0	2

() indicate grade 3 or higher.

Supplementary Table 2: Sorafenib trough plasma concentrations (g/mL)

Dose levels	Patient #	C1D2 Predose	C1D8/9 Predose	C1D10 Predose	C1D15/16 Predose	Average of Cmin,ss on C1D8, C1D10 and C1D15
**Level 1** Sorafenib 200 mg qd and Riluzole 100 mg BID	#1	0.368	3.661	1.177	1.869	2.236
	#2	1.422	2.487	1.845	1.943	2.092
	#3	*0.686*	*4.934*	*4.771*	*5.905*	–
	#4	1.273	4.755	1.972	1.493	2.74
	**Mean (SD)**	**1.021 (0.57)**	**3.634 (1.134)**	**1.665 (0.427)**	**1.768 (0.241)**	**2.356**
	**Median**	**1.273**	**3.661**	**1.845**	**1.869**	
**Level 2** Sorafenib 200 mg BID and Riluzole 100 mg BID	#5	0.968	6.487	5.173	15.39	9.017
	#6	4.68	2.817	3.305	4.595	3.572
	#7	1.467	3.284	3.37	3.674	3.443
	#8	6.093	14.27	9.022	10.145	11.146
	#9	4.16	2.359	*4.296*	3.394	3.35
	#10	2.103	6.376	3.472	3.528	4.459
	**Mean (SD)**	**2.958 (2.132)**	**5.932 (4.461)**	**4.868 (2.449)**	**6.788 (4.938)**	**5.752**
	**Median**	**2.103**	**4.83**	**3.472**	**4.134**	
**Level 3** Sorafenib 400 mg AM, 200 mg PM and Riluzole 100 mg BID	#11	1.831	2.221	2.879	3.456	2.852
	#12	1.652	4.327	4.436	11.044	6.602
	#13	2.829	N/A	N/A	N/A	–
	#14	1.011	1.581	3.211	2.329	2.374
	#15	2.554	9.444	8.198	N/A	8.821
	#16	2.742	3.71	4.756	9.444	5.97
	#20	2.004	6.577	N/A	N/A	6.577
	#21	3.555	2.844	5.196	3.063	3.701
	#22	2.167	2.456	1.924	2.218	2.199
	#23	3.762	12.392	11.684	7.476	10.517
	#24	3.719	7.478	7.774	9.122	8.125
	#25	2.648	7.868	7.898	12.054	9.273
	#26	2.086	4.303	3.96	6.526	4.93
	#27	3.369	6.361	10.4	6.494	7.752
	#28	1.232	4.21	N/A	N/A	4.21
	#30	1.423	10.251	10.471	8.984	9.902
	#31	1.951	3.889	4.149	5.882	4.64
	#32	1.286	4.917	3.243	1.061	3.074
	#33	6.307	8.15	10.666	9.29	9.369
	#34	1.117	*3.989*	4.55	4.54	4.545
	#35	2.129	0.789	0.149	<LOD	–
	#36 (Ex)	0.51	2.418	2.861	*2.734*	2.671
	**Mean (SD)**	**2.294 (1.259)**	**5.547 (3.042)**	**6.014 (3.171)**	**6.436 (3.391)**	**5.905**
	**Median**	**2.108**	**4.327**	**4.653**	**6.51**	
**Level 4** Sorafenib 400 mg BID and Riluzole 100 mg BID	#17	5.491	8.255	16.769	3.628	9.551
	#18	5.406	6.126	9.452	3.95	6.509
	#19	2.184	3.458	4.998	6.493	4.983
	**Mean (SD)**	**4.360 (1.885)**	**5.946 (2.404)**	**10.406 (5.943)**	**4.690 (1.569)**	**7.014**
	**Median**	**5.406**	**6.126**	**9.452**	**3.95**	

Data underlined was excluded from the statistical analysis since the patients did not follow the dose compliance based on the flow sheet records. N/A, no sample was collected on the time point. <LOD, below limit of detection.

Supplementary Table 3: Plasma trough steady state concentrations (Cmin,ss), expressed as the geometric mean, of riluzole and sorafenib at different dose levels

Dose Levels	Riluzole Cmin,ss (ng/mL)	Sorafenib Cmin,ss (μg/mL)	Sorafenib accumulation factor
	Geometric mean, Riuzole (CV%)	Geometric mean, Sorafenib (CV%)	
**Level 1 (*n* = 4)**	34.93 (64)	2.34 (14)	2.31
**Level 2 (*n* = 6)**	48.84 (65)	5.01 (60)	1.94
**Level 3 (*n* = 22)**	79.72 (153)	5.25 (47)	2.58
**Level 4 (*n* = 3)**	25.98 (25)	6.77 (33)	1.61

Cmin,ss was calculated as the average trough level on D8, D10 and D15. Accumulation factor was calculated as the ratio of average Css of D8, D10 and D15 to D2. Abbreviation: CV: Coefficient of Variance.

Supplementary Table 4: Riluzole trough plasma concentrations (ng/mL)

Pt ID	Riluzole concentration (ng/mL)			
	D2 Predose	D8/9 Predose	D10 Predose	D15 Predose
**Dose Level 1: Sorafenib 200 mg qd and Riluzole 100 mg BID**				
#1	3.33	36.89	8.9	24.17
#2	62.45	78.14	68.6	75.67
#3	57.32	62.61	51.11	36.97
#4	10.74	22.29	15.26	13.85
Geomean (CV)	18.92 (92)	44.79 (50)	26.27 (80)	31.11 (72)
**Dose Level 2: Sorafenib 200 mg BID and Riluzole 100 mg BID**				
#5	35.07	83.1	115.98	170.15
#6	95.29	69.82	62.36	101.4
#7	45.1	35.73	39.19	48.51
#8	42.18	28.19	36.03	35.45
#9	142.56	47.99	40.55	30.81
#10	26.84	22.33	20.11	36.43
Geomean (CV)	58.35 (82)	42.93 (50)	45.97 (68)	56.72 (79)
**Dose Level 3: Sorafenib 400 mg AM, 200 mg PM and Riluzole 100 mg BID**				
#11	28.71	17.87	14.45	20.22
#12	73.18	116.11	127.21	124.67
#13	32.02	N/A	N/A	N/A
#14	30.21	43.44	47.08	49.06
#15	47.71	32.96	61.84	N/A
#16	53.1	32.87	47.42	47.37
#20	56.61	90.92	N/A	N/A
#21	37.24	43.12	39.41	66.2
#22	64.09	47.28	52.51	68.14
#23	19.62	18.2	13.19	7.02
#24	58.86	67.09	60.1	76.1
#25	66.84	96.47	89.82	85.03
#26	18	83.41	76.9	92.65
#27	41.51	62.44	69.2	71.35
#28	55.95	81.2	N/A	N/A
#30	47.37	455.25	142.35	60.98

#31	127.44	149.76	128.03	135.37
#32	40.63	57.58	57.99	80.05
#33	119.96	54.31	48.4	40.12
#34	295.14	815.72	703.83	882.52
#35	250.15	6.14	<LOD	<LOD
#36	253.77	194.16	1368.52	1014.41
Geomean (CV%)	59.88 (97)	66.01 (109)	77.34 (175)	67.50 (119)
**Dose Level 4: Sorafenib 400 mg BID and Riluzole 100 mg BID**				
#17	47.38	31.53	37.2	33.44
#18	36.46	25.95	38.88	7.23
#19	14.16	15.07	18.41	30.81
**Geomean (CV%)**	**29.03 (52)**	**23.10 (35)**	**29.86 (36)**	**19.53 (61)**
**Total Geomean (CV%)**	**47.15 (71)**	**52.57 (112)**	**55.83 (103)**	**51.47 (177)**
**Median**	**47.37**	**51.15**	**52.51**	**49.06**

Data are represented as geometric mean (% coefficient of variation). Abbreviations: N/A: sample unavailable; LOD: limit of detection.

Supplementary Table 5: Immunohistochemistry and Western Blot results and quantification

Pt ID	IHC Results			Western Blot Results			Best Response	Tumor Type
	GRM1 Pre/Post	BCL-2 Pre/Post	BIM Pre/ Post	pERK/total ERK Pre/Post	pAKT/Total AKT Pre/Post	pCRAF/ Total CRAF Pre/Post		
2	**1.84/ 0.00091**	47.61/ 45.24	95.58/ 45.24	N/A*	N/A	N/A	PD	Melanoma
23	**58.17/ 27.2**	**42.45/ 30.06**	**46.32/ 87.07**	N/A	N/A	N/A	PD	Sarcoma
26	**34.02/ 15.9**	N/A	N/A	N/A	N/A	N/A	SD	Melanoma
27	60.05/ 66.36	17.16/ 68.23	37.15/ 58.85	**0.98 ± 0.1/ 0.19 ± 0.09**	**1.25 ± 0.09/ 0.17 ± 0.01**	1.04 ± 0.09/ 8.96 ± 1.3	SD	Melanoma
30	N/A	**46.96/ 33.4**	2.02/ 0.11	0.58 ± 0.08/ 0.64 ± 0.3	0.85 ± 0.06/ 1.85 ± 0.09	N/A	PD	Melanoma
31	72.93/ 73.04	21/ 44.74	**20.67/ 28.66**	**0.48 ± 0.09/ 0.21 ± 0.06**	**0.62 ± 0.004/ 0.18 ± 0.03**	2.93 ± 0.09/ 2.2 ± 0.08	NE	Melanoma
32	N/A	N/A	N/A	**4.65 ± 1.2/ 2.01 ± 0.9**	**0.44 ± 0.03/ 0.19 ± 0.1**	2.69 ± 1.2/ 0.02 ± 0.1	SD	Melanoma
33	**87.44/ 59.26**	32.92/ 33.8	**2.8/ 12.93**	0.72 ± 0.05/ 0.82 ± 0.04	**0.97 ± 0.08/ 0.78 ± 0.06**	1.02 ± 0.6/ 1.38 ± 1.01	PR	Pancreas
36	N/A	N/A	N/A	**1.6 ± 0.08/ 0.02 ± 0.001**	**0.04 ± 0.001/ 0.03 ± 0.01**	0.02 ± 0.01/ 0.03 ± 0.01	SD	Endometrial

Intensities of specific bands from Western immunoblots of protein lysates prepared from available paired pre- and post-treatment patient specimens and probed with pERK, total ERK, pAKT, total AKT, and pCRAF, total CRAF. Patient responses are listed on the last column. The values of the quantifications were the mean ± SD of three independent experiments. Values bolded were statistically significant ($p < 0.05$). *Insuffcient tissue.
